# Work motivation and its effects on organizational performance: the case of nurses in Hawassa public and private hospitals: Mixed method study approach

**DOI:** 10.1186/s13104-019-4255-7

**Published:** 2019-04-08

**Authors:** Ababe Tamirat Deressa, Getachew Zeru

**Affiliations:** 1 School of Nursing, College of Medicine and Health Sciences, Hawassa University, Hawassa, Ethiopia; 2 African Institute of Governance & Development, Ethiopian Civil Service University, Addis Ababa, Ethiopia

**Keywords:** Perception of motivation, Effects, Nurses, Organizational performance

## Abstract

**Objective:**

The main objective of the study was to assess level of motivation, how nurses perceived work motivation and its effects on organizational performance among nurses working in Hawassa public and private hospitals.

**Results:**

It was found that majority (64.1%) of the nurses perceived motivation as motivators. Getting prospective encouragement, recognition and financial incentives were the main descriptions the nurses gave to motivation. Increased work performance, job satisfaction, good team spirit, patient satisfaction and job attachment were the reported effects of nurses’ motivation.

**Electronic supplementary material:**

The online version of this article (10.1186/s13104-019-4255-7) contains supplementary material, which is available to authorized users.

## Introduction

Rahimic [1] defined motivation as an individual’s level of readiness to perform an action and it comprises all factors that influence, intensify and organize human behavior. Motivation in the work context is expressed as an individual’s degree of willingness to exert and maintain an effort towards organizational goals. Employees have different competing needs that are driven by various motivators. Therefore, to maximize organizational performance, organization and its managers should understand what really motivates the employees [2].

Over 50% of health workers in Benin equate motivation with prospective encouragement or retrospective compensation that is considered as what makes them work better. Majority of them considered motivation as a “motivator”, i.e. an incentive, and not as a state of mind. In Kenya, one-fifth understands motivation as encouragement, however, there is a larger share of health workers who refer to that intrinsic state of willingness and pleasure to do one’s work [3].

Motivation and job satisfaction were both significantly associated with turnover intention [4]. Low motivation has a negative impact on the performance of individual health workers, facilities and the health system as a whole [5, 6].

The evidence that 60.9% nurses in Turkey reported having intention to quit the present workplace within 1 year was secondary to lack of job satisfaction or motivation by majority of them [7]. On the other hand, it is researched in Istanbul as administrator being informed about the motivation of their employees boosts the morals of the employees [8]. Physicians of primary health facilities in Pakistan reported that they leave the organization if they are not motivated [9].

Health workers with higher levels of motivation and job satisfaction in Ghana were less likely to have intentions to leave their current health facilities [4]. Study on Ethiopian public health workers also evidenced as overall performance of health workers is negatively impacted by low levels of health worker motivation and job satisfaction. Job satisfaction is to be important because of its hypothesized association with internal motivation and overall job performance [10].

The purpose of the study was do assess motivation level of the nurses, how the nurses perceived work motivation and its effects on organizational performance that can contribute for improving work motivation in nursing service leadership.

## Main text

### Methods

This study was conducted with mixed method study design. From the strategies of mixed method, concurrent triangulation was considered to triangulate the qualitative with the quantitative data. Purposive sampling was applied to select the hospitals based on the number of nurses they employed. Accordingly, two hospitals (Hawassa university comprehensive and specialized hospital from public, and Alatyon hospital from private hospitals were selected). A total sample of 241 nurses were selected and the response rate was 91.3%. After proportional allocation was computed to each ward in the hospital, simple random sampling with lottery technique was used to select nurses from each ward. Again, Purposive sampling was used to select key informant interviewees (head ward nurse, hospital manager and patients) for the qualitative approach. Different study subjects (e.g. hospital manager and patients) were included for the objective of identifying effects motivation on organizational performance. Hence, seven nurses on position (ward head, matron or CEO (chief executive officer) of nursing service administration), two hospital managers and six patients were selected.

Self-administered questionnaire was used for collecting data for the quantitative approach. Pre-test was conducted in unselected public hospital (Adare hospital). The questionnaire adopted from [3, 11, 12] and modified according to objectives of the study. The multidimensional work motivation scale (MWMS) was applied to measure the work motivation. From 18 options of Multidimensional Work motivation scale (MWMS) to the question ‘Why do you or would you put efforts into your current job?’ the first fifteen (A-O) measures motivation. Each option is scored out of seven starting from ‘Not at all’ (1) to ‘Completely’ (7) answer to the option. Thus, starting from ‘moderately’ (4 point for each) and a total sum of ≥ 60 out of 105 was considered as having work motivation. The qualitative check list was also designed by adopting from different studies. Qualitative data was collected through face to face interview and sound record at work place (in hospitals) after getting oral and written consent from the respondents with involvement of the both principal and co-authors. In addition, field notes were taken during data collection. Interview was accomplished within 20–30 min and data saturation determined the last sample. No relationship was established prior to study commencement with the participants.

For the quantitative data, data collected was coded and entered into EpiData 3.1. After this, it was exported to SPPSS 22 where data cleaning and analysis was conducted. Moreover, sum of scores for multidimensional work motivation scale was done with this software and recoding the variable as ‘motivated and not motivated’ was created on this base. The records were transcribed and coded with two coders. Then, themes were identified depending on repeated expressions (Additional file 1: Table S1) in the transcripts and thematic analysis with narrative report writing was applied for qualitative data. The transcripts were also returned to participants for checking.

### Results

#### Socio-demographic characteristics

From the total 241 distributed questionnaires, 220 (giving 91.3% of response rate) were returned and used for analysis of the quantitative data. Majorities (51.4%) of the respondents were male with mean age of 27.55 (± 4.039 SD). The mean service year for the nurses were 5.55(± 4.131 SD) and majority (60.0%) of them served less than 5 years. Largest share (46.4%) and 74.1% of the nurses were orthodox religion followers and B.Sc. degree holders respectively (Additional file 2: Table S2). Four of the nurses responded for the interview were female and three of them were male. The two hospital managers were male. Moreover, four selected patients were female and two of them were male.

#### Perception towards work motivation

Large number of nurses (49.1%) stated as motivation is prospective encouragement for performance followed by getting recognition (39.5%) and financial incentives (37.3%). Nurses from the public hospital largely described motivation in terms of prospective encouragement (49.3%). On the other hand, getting award (81.8%) was the dominant descriptions of motivation from the private hospital (Table 1).

**Table 1:** Perception towards work motivation among nurses, April, 2017 (n = 220)

Perceived definition of motivation	Category of the hospital		Total^a^ (%)
	Public^a^ (%)	Private^a^ (%)	
*Prospective encouragement*			
Yes	103 (49.3)	5 (45.5)	108 (49.1)
No	106 (50.7)	6 (54.5)	112 (51.9)
*Retrospective compensation*			
Yes	20 (9.6)	0 (0.0)	20 (9.1)
No	189 (90.4)	11 (100.0)	200 (90.9)
*Having means and materials to work*			
Yes	39 (18.7)	4 (36.4)	43 (19.5)
No	170 (81.3)	7 (63.6)	177 (80.5)
*Getting recognition*			
Yes	79 (37.8)	8 (72.7)	87 (39.5)
No	130 (62.2)	3 (27.3)	133 (60.5)
*Getting awards*			
Yes	63 (30.1)	9 (81.8)	72 (32.7)
No	146 (69.9)	2 (18.2)	148 (67.3)
*Financial incentives*			
Yes	74 (35.4)	8 (72.7)	82 (37.3)
No	135 (64.6)	3 (27.3)	138 (62.7)
*Willingness to do one’s work*			
Yes	53 (25.4)	2 (18.2)	55 (25.0)
No	156 (74.6)	9 (81.8)	165 (75.0)
*Pleasure to do one’s work*			
Yes	55 (26.3)	3(27.3)	58 (26.4)
No	154 (73.7)	8 (72.7)	162 (73.6)
*Respecting others and your self*			
Yes	2 (1.0)	0 (0.0)	2 (0.9)
No	207 (99.0)	11 (100.0)	218 (99.1)

^a^Frequency in number

Similarly, from the qualitative data, nurse perceived motivation as it is doing one’s work with full interest, doing good things and sacrificing yourself as much as possible for others.

For me, motivation is doing your own work without any enforcement, that mean with no interference of anyone, by yourself after accepting your profession; but first the person should accept his own profession and I, myself, love my profession. For me, motivation is these all (27 years old ward head nurse with 5 years work experience from public hospital).

Actually I’m not this much good in narration! But, thinking empathically for people with sense of ‘had it been me’ and when the person does good, may be difficult always, but with all possible extents; this is motivation (28 years old head ward nurse with 4 years of service from the private hospital).

From multidimensional work motivation scale (MWMS) used to measure the motivation of nurses, doing the work because the job is part of their life was scored highly with mean of 3.78 and mode of 4 (Table 2). The mean score for the summation of MWMS score was 53.13 (± 20.16 SD). Majority (61.4%) of them, from which nurses from the public hospital account for great number, were not or less motivated (scored < 60/105) whereas only 85(38.6%) of them were found to be motivated (scored ≥ 60/105) (Additional file 3: Figure S1).

**Table 2:** Multidimensional work motivation scale (MWMS) for nurses’ motivation, April, 2017

Variables for MWMS	Mean	Standard deviation	Median	Mode
Because I derive much pleasure from learning new things	3.79	1.758	4.00	5
For the satisfaction I experience from taking on interesting challenges	3.76	1.717	4.00	2
For the satisfaction I experience when I am successful at doing difficult tasks	3.78	1.778	4.00	2
Because it has become a fundamental part of who I am	3.76	1.848	4.00	2
Because it is the way which I have chosen	3.46	1.758	3.00	3
Because this job is a part of my life	3.80	1.840	4.00	4
Because this is the type of work I chose to do to attain a certain lifestyle	3.60	1.781	3.50	2
Because it is the type of work I have chosen to attain certain important objectives	3.65	1.768	3.50	2
Because I chose this type of work to attain my career goals	3.61	1.804	3.00	2
Because I want to succeed at this job	3.58	1.820	3.00	2
Because I want to be very good at this work, otherwise I would be very disappointed	3.28	1.770	3.00	2
Because I want to be a “winner” in life	3.56	1.856	3.00	2
Because it is the only source of income for me	3.59	1.941	3.00	2
Because it allows me to earn additional income	2.97	1.717	2.50	2
Because this type of work provides me with security	2.95	1.663	3.00	2

#### Effects of motivation on organizational performance

**Fig. 1:**
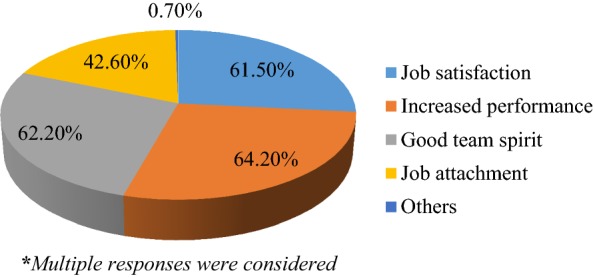
Effects of work motivation on organizational performance, April, 2017

From 219 participants who responded to effect of work motivation, 67.9% (147) of them believed that their motivation has effect on their organizational performance and 32.9% (72) of them responded as their motivation has no effect on their organizational performance. The majority (64.2%) stated increased work performance followed by good team spirit (62.2 as the effect of their motivation (Fig. 1).

Though some report from the patient in case of public hospital is different, the interview result also agrees with the above quantitative finding.

It is said that ‘doctor treats disease and nurse treats patient’. So, if nurses are motivated; patient will be satisfied, heals quickly and hospital stay will be decreased. Even work that may take long time can be accomplished within short period (28 years old head ward nurse and head of nursing service administration from the public hospital).

Motivated or satisfied servant provides satisfying services. Thus, the foremost result of nurses’ motivation is patients’ satisfaction (31 years old nurse with 10 years of service from private hospital).

Public hospital manager stated that “nurses’ motivation can bear many effects on the organizational performance. To say some of them: infection prevention will be practiced in efficient way and this can contribute to reduction of morbidity and mortality in the hospital. Moreover, development of complications from health care service can be significantly controlled if we have motivated nurses. These all result in patients’ satisfaction.

The private hospital manager also said that “when nurses are motivated; they become punctual, serve the patient quickly, give standardized care and create good team spirit within the organization. That is why our customer admires our nurses by saying ‘your nurses, your nurses…’ we hear this”.

In line with the above, patient from the private hospital realized this report. A 25 years old patient who was admitted 5 days back responded; “Here it is good; they follow you timely, give medication on time, make bed daily and appear immediately when you call them. I will return here in the future if I need health care and also invite others”. Clean, made and attractive bed was also observed. But, medication sheet that is not uniformly distributed and maintained in the private hospital near the patient’s bed was actively working in the public hospital.

In public hospitals some patient responded getting timely medication and follow up from nurses. Some beds were observed naked without linen, not made and this was totally different from neat and made bed in the private hospital. Though her bed was made clean, a 23 years old patient who was admitted before 14 days of the data collection period complained “nurses sometimes don’t come and ignore me when I need and call them”.

### Discussion

Mainly motivators were perceived as motivation in this study. This is in line with study in Benin [3] that indicated majority of nurses equated motivation with some motivators. Few nurses than report from Benin stated as motivation is having means and materials to work in this study. Socio-economic difference might contribute for this variation. It is known that Ethiopia is the developing country whose most people didn’t achieve their basic needs yet. This could be the reason why nurses in this study described motivation in the view of financial benefit than its work and work related expression. But, in line with the study of Benin; majority of the nurses explained motivation as motivator, not as set of mind. Thus, less number of nurses stated motivation as willingness or pleasure to do work. This disagree with the finding from Kenya in which larger share described motivation as willingness and pleasure to do one’s work [3]. The study design used and socio-economic difference in which nurses in this setup are paid less than those countries may contribute for this variation.

The qualitative report that says “For me, motivation is doing your own work without any enforcement, that mean with no interference of anyone after accepting your profession” partly agrees with definition that has been stated by indicating motivation as individual’s degree of willingness to exert and maintain an effort towards organizational goals [13]. Since issue of motivation is highly emphasized in professionals of social sciences, lack of optimal awareness about motivation can contribute to the perception of nurses about motivation in this way.

Similar to study in Addis Ababa, this study identified as majority of nurses are not motivated especially in the public sector [14]. This is also supportable with finding from South Africa that identified as overall doctors were dissatisfied in their work motivation [15]. Difficulty of getting vital things as needed and absence of practicing motivation by the public organization in developing countries may be the reason behind of these scenarios.

The majority of nurses in this study stated increased work performance followed by job satisfaction to be effect of their motivation. Similarly, these were the dominantly reported effects in Benin study [3]. Again, the patient satisfaction from the performance was described by nurses and hospital managers in the interview. Observation result also witnessed this finding that the more motivated private hospital’s nurses give better care such as comforting and making the patient’s bed.

### Conclusions

Majority of nurses perceived motivation as motivators. Getting prospective encouragement, recognition and financial incentives were the main descriptions the nurses gave to motivation. Very few of them described motivation in terms of non-financial incentives. Greater part of them, from which nurses of the public hospital accompany the large share, were not or less motivated. Increased work performance, job satisfaction, good team spirit, patient satisfaction and job attachment were the identified effects of nurses’ motivation.

## Limitations

- The cross sectional study design may not guarantee the exact assessment of cause-effect relationship.
- Not using software for qualitative data analysis may not have strong analysis output.
- Scarcity of resources from the public hospital might bias the observation data collection.

## Additional files

**Additional file 1: Table S1.** Themes identified for thematic analysis.

**Additional file 2: Table S2.** Socio-demographic characteristics of the nurse respondents, April, 2017.

**Additional file 3: Figure S1.** Level of motivation for nurses, April, 2017.

## Abbreviations

CEO: Chief Executive Officer
MWMS: Multidimensional Work Motivation Scale
SD: standard deviation
SPPSS: Statistical Package for Social Sciences
