# Supplementary material for: Work motivation and its effects on organizational performance: the case of nurses in Hawassa public and private hospitals: Mixed method study approach
Source: BMC Res Notes. 2019 Apr 8;12:213. doi: 10.1186/s13104-019-4255-7 (PMC6454626; doi:10.1186/s13104-019-4255-7)
Supplement: Supplementary file 1 — Additional file 1: Table S1. Themes identified for thematic analysis. [file 13104_2019_4255_MOESM1_ESM.docx]

**Table S1:** Themes identified for thematic analysis

| **Themes** | **Frequency of repetition** |
| --- | --- |
| Love of profession | 36 times |
| Absence of enforcement | 27 times |
| Satisfying services | 36 times |
| Doing good | 36 times |
| Good team spirit | 27times |
| Standardized care | 24 times |
| Good time management | 30 times |
