# Supplementary material for: Work motivation and its effects on organizational performance: the case of nurses in Hawassa public and private hospitals: Mixed method study approach
Source: BMC Res Notes. 2019 Apr 8;12:213. doi: 10.1186/s13104-019-4255-7 (PMC6454626; doi:10.1186/s13104-019-4255-7)
Supplement: Supplementary file 2 — Additional file 2: Table S2. Socio-demographic characteristics of the nurse respondents, April, 2017. [file 13104_2019_4255_MOESM2_ESM.docx]

**Table S2: Socio-demographic characteristics of the nurse respondents, April, 2017**

| **Characteristics** | | **Frequency** | **Percent** |
| --- | --- | --- | --- |
| **Sex** | Male | 113 | 51.4 |
|  | Female | 107 | 48.6 |
| **Age** | <25 | 72 | 32.7 |
|  | >25 | 148 | 67.3 |
| **Religion** | Protestant | 87 | 39.5 |
|  | Orthodox | 102 | 46.4 |
|  | Muslim | 15 | 6.8 |
|  | Catholic | 5 | 2.3 |
|  | Others***** | 11 | 5.0 |
| **Educational status** | Diploma | 55 | 25.0 |
|  | Bsc degree | 163 | 74.1 |
|  | Master’s degree | 2 | 0.9 |
| **Total Service year** | <5 | 132 | 60.0 |
|  | 6-10 | 65 | 29.5 |
|  | **>**10 | 23 | 10.5 |
