# Supplementary material for: Work motivation and its effects on organizational performance: the case of nurses in Hawassa public and private hospitals: Mixed method study approach
Source: BMC Res Notes. 2019 Apr 8;12:213. doi: 10.1186/s13104-019-4255-7 (PMC6454626; doi:10.1186/s13104-019-4255-7)
Supplement: Supplementary file 3 — Additional file 3: Figure S1. Level of motivation for nurses, April, 2017. [file 13104_2019_4255_MOESM3_ESM.docx]

**Figure S1: Level of motivation for nurses, April, 2017**
